# Investigation of the efficacy of mycotoxin-detoxifying additive on health and growth of newly-weaned pigs under deoxynivalenol challenges

**DOI:** 10.5713/ajas.20.0567

**Published:** 2020-10-20

**Authors:** Debora Muratori Holanda, Sung Woo Kim

**Affiliations:** Department of Animal Science, North Carolina State University, Raleigh NC 27695, USA

**Keywords:** Adsorbent, Deoxynivalenol, Enzyme, Gut Health, Pig, Probiotic

## Abstract

**Objective:**

This study evaluated the effects of feeding diets naturally contaminated with deoxynivalenol (supplemental 2 mg/kg) on health, growth, and the effects of a mycotoxin-detoxifying additive in newly-weaned pigs.

**Methods:**

Thirty-six pigs (27 day-old) were housed individually and assigned to 3 treatments for 5 weeks: CON (diet containing minimal deoxynivalenol), MT (diet with supplemental 1.9 mg/kg of deoxynivalenol), and MT+D (MT + mycotoxin-detoxifying additive, 0.2%, MegaFix, ICC, São Paulo, Brazil). The mycotoxin-detoxifying additive included bentonite, algae, enzymes, and yeast. Blood was taken at week 2 and 5. Jejunal tissue were taken at week 5. Data were analyzed using the MIXED procedure of SAS.

**Results:**

Pigs fed MT+D tended to have decreased (p = 0.056) averaged daily feed intake during week 1 than MT. At week 2, serum aspartate aminotransferase/alanine aminotransferase in MT tended to be lower (p = 0.059) than CON, whereas it was increased (p< 0.05) for MT+D than MT, indicating hepatic damages in MT and recovery in MT+D. Pigs fed MT had lower (p<0.05) blood urea nitrogen/creatinine than CON, supporting hepatic damage. At week 5, pigs fed MT tended to have reduced (p = 0.079) glucose than CON, whereas it was increased (p<0.05) for MT+D than MT, indicating impaired intestinal glucose absorption in MT, which was improved in MT+D. Pigs fed CON tended to have increased (p = 0.057) total glutathione in jejunum than MT, indicating oxidative stress in MT. Pigs fed MT+D had a reduced (p<0.05) proportion of Ki-67-positive cells in jejunum than MT, indicating lower enterocyte proliferation in MT+D.

**Conclusion:**

Feeding supplemental 1.9 mg/kg of deoxynivalenol reduced growth and debilitated hepatic health of pigs, as seen in leakage of hepatic enzymes, impaired nitrogen metabolism, and increase in oxidative stress. The mycotoxin-detoxifying enhanced hepatic health and glucose levels, and attenuated gut damage in pigs fed deoxynivalenol contaminated diets.

## INTRODUCTION

Mycotoxins are secondary metabolic products of fungi with toxigenic effects in other living species. Worldwide, about 88% of animal feed and feedstuffs are contaminated with at least one mycotoxin [1]. *Fusarium* toxins are the most prevalent, where deoxynivalenol ranks at first with 64% of occurrence, followed by fumonisins and zearalenone [1].

In pigs chronically fed deoxynivalenol contaminated diets, it is observed increased expression of interleukin 8 and glutathione peroxidase [2]. Deoxynivalenol also has shown impairment in the translation of mRNA that may ultimately affect cell proliferation, immune response, development, death, [3–5] oxidative stress, and reduction in feed intake and growth of pigs [6,7]. Pigs challenged with naturally contaminated diets with deoxynivalenol showed impaired gut health by reducing enterocyte proliferation and intestinal surface, resulting in compromised weight gain [8].

The chemical properties of deoxynivalenol make it difficult to be adsorbed or neutralized by mycotoxin-detoxifying additives. Thus, microorganisms, inorganic compounds, aluminosilicates, and yeast-based products are tested aiming to reduce deoxynivalenol toxicity [9–12] as in the mycotoxin-detoxifying additive tested in the current study. The aluminosilicates, such as the bentonite, have proven ability to adsorb mycotoxins based on the electrical charge of molecules [13,14]. Similarly, autolyzed yeast has demonstrated higher specificity for deoxynivalenol adsorption in comparison to mineral adsorbents [13,15]. At the same time, the extensive use of *Saccharomyces cerevisiae* (*S. cerevisiae*) animal species with neutral or beneficial outcomes, and its availability turns its utilization feasible and positive to the livestock feed industry [16]. Of interest, *S. cerevisiae* was able to reduce inflammation and oxidative stress and to increase cell survivability in deoxynivalenol challenged pigs [17]. In association with those, the tested mycotoxin-detoxifying additive has an enzyme complex composed of esterase, epoxide-reductase, and peptidase can detoxify deoxynivalenol [18], and other *Fusarium* toxins [19]. The algae powder derived from *Lithothamnium calcareum* (*L. calcareum*) has shown *in vitro* adorability towards zearalenone [20] and both *in vitro* and *in vivo* (broilers) toward aflatoxin B1 [21] but no studies have investigated its efficacy towards deoxynivalenol to date. Thus, the use of mycotoxin detoxifiers with multiple components, targeting a synergistic effect, is preferred to boost detoxification effects [12,22].

After analyzing publications on deoxynivalenol challenges in pigs, it was observed that each supplemental mg/kg of the mycotoxin in the feed would result in an 8% decrease in animal growth [23]. It is hypothesized that deoxynivalenol supplemented at 2 mg/kg would impair the health and growth of newly-weaned pigs and that using a mycotoxin-detoxifying additive including adsorbents (aluminosilicates and yeast cell wall - β-glucans), antioxidants, and immune modulators (algae and yeast as probiotic), and components to inactivate mycotoxins (enzymes), would attenuate the negative impacts of deoxynivalenol to newly-weaned pigs. The objective of this study was to evaluate the effects feeding diets naturally contaminated with deoxynivalenol (supplemental 2 mg/kg) on health and growth and the efficacy of a mycotoxin-detoxifying additive in mitigating such effects in newly-weaned pigs.

## MATERIALS AND METHODS

The Institutional Animal Care and Use Committee at North Carolina State University (Raleigh, NC, USA) reviewed and approved a protocol for conducting this study at the North Carolina State University Metabolism Educational Unit (Raleigh, NC, USA).

### Animals and diets

Upon weaning at 27 d of age (d 0), 36 barrows and gilts (8.21 ±0.54 kg) were individually assigned to 3 dietary treatments based on a randomized complete block design. Blocks were sex (male and female) and initial body weight (light, medium, and heavy). Pigs were individually housed in pens with metal screen floors with individual nipple drinkers and feeders. Each pig was fed one of the 3 dietary treatments for 34 days: i) a control diet with minimal deoxynivalenol contamination (CON), ii) CON with a supplemental 1.9 mg/kg of deoxynivalenol (MT), and iii) MT + mycotoxin-detoxifying additive (MegaFix, ICC, São Paulo, Brazil) at 0.2% (MT+D). Based on the safety of yeast additives in swine diets (over 10 years) [16], and the effect of similar mycotoxin detoxifiers (adsorbent and yeast-based products) have been previously tested [8,24], this study was not to test the effects of mycotoxin-detoxifying additive in a control diet but only in a diet with mycotoxin [12]. The CON diet was formulated with “clean corn dried distillers grains with solubles (DDGS)”, minimal mycotoxin contamination, whereas MT was formulated with “deoxynivalenol contaminated corn DDGS” (Table 1). The nutrient requirements suggested by National Research Council (NRC, 2012) [25] was used to formulat experimental diets (Table 2). Diets followed a 3-phase feeding program: phase 1, during 1 week; phase 2, from week 2 until week 3; and phase 3, from week 4 until week 5. Phase 3 was shortened in 1 day for better arranging sampling schedule, so week 5 lasted for 6 days.

Growth performance was recorded weekly by measuring body weight of pigs and feed consumption by pigs to obtain averaged daily gain, averaged daily feed intake, and to calculate gain to feed ratio. Fecal score of pigs was recorded on d 5, and at the end of week 1 and week 2 by ranking feces on a scale from 1 to 5 by a single evaluator. Feces considered in level 1 on the proposed scale are considered “normal” and have increasing water content until reaching level 5. In level 2, feces have increased water content but the characteristic shape of feces is maintained. In level 3, feces start to loose their characteristic shape, frequently with liquid and solid components mixed. In level 4, feces become viscous and pasty, with no vertical structure. In level 5, feces are liquid and do not have any consistency. At the end of week 2 and week 5, the external jugular vein was punctured using needles (0.8×32 mm; Eclipse, Becton Dickinson Vacutainer Systems, Franklin Lakes, NJ, USA) to obtain 10 mL of blood samples into serum blood collection tubes (Becton Dickinson Vacutainer Systems, USA). Blood samples were allowed to clot for 4 hours at room temperature, blood samples were centrifuged at 1,500×g at 4°C for 15 minutes (5811F, Eppendorf, Hamburg, HH, Germany) to obtain blood serum. Blood serum samples were stored at −80°C freezer (812660-760, Thermo Fisher Scientific, Waltham, MA, USA) in 1.5 mL microtubes (Fisherbrand, Fisher Scientific, Hampton, NH, USA) until laboratory analyses.

By the end of week 5, a penetrating captive bolt was used to desensitize pigs and they were euthanized by immediate vena cava section for the collection of mid-jejunal mucosa and tissue. A mid-jejunal section of 15 cm was scrapped with a clean histological slide to obtain mid-jejunal mucosa [12]. Mucosa samples were stored 1.5 mL microtubes at −80°C until laboratory analyses. A second tissue section of 5 cm from mid-jejunum was fixed in 10% buffered formaldehyde and stored at room temperature.

### Assay procedures

Diets from each experimental and within each phase were sampled from 9 different locations, a total of 2 kg per diet. Sub-samples of 200 g of each diet and phase were sent to the North Dakota State University Veterinary Diagnostic Laboratory (Fargo, ND, USA) for mycotoxin analysis, and 300 g of each diet were sent to the North Carolina Department of Agriculture (Raleigh, NC, USA) for proximate analysis. The CON had detectable deoxynivalenol levels because corn DDGS used in CON formulation had deoxynivalenol contamination. Nevertheless, MT had 1.9 mg/kg more deoxynivalenol than CON, close to the planned concentration of 2 mg/kg (Table 3).

Serum samples were submitted for a biochemical profile at Antech Diagnostic Laboratory (Cary, NC, USA). Antioxidant status and immune markers were evaluated in mid-jejunal mucosa by quantifying protein carbonyls (STA-310, Cell Biolabs, Inc., San Diego, CA, USA), malondialdehydes (STA-330, Cell Biolabs, Inc., USA), total glutathione (STA-312, Cell Biolabs, Inc., USA), tumor necrosis factor-alpha (PTA00, R&D Systems, Inc., Minneapolis, MN, USA), and interleukin-8 (P8000, R&D Systems, Inc., USA). The manufacturer’s manual for each kit was followed in the laboratory assays of protein carbonyls, malondialdehydes, and total protein according to procedures described by Zhao and Kim [26]. Measurements of total glutathione, tumor necrosis factor-α, and interleukin-8 followed the procedures as described by Holanda et al [24].

Transversal sections of 0.5 cm were transferred to 70% ethanol after 14 days and sent to the North Carolina State University Histopathology Laboratory (College of Veterinary Medicine, Raleigh, NC, USA), where samples were included in paraffin, microtomed, and stained for Ki-67 antigen by immunohistochemistry before assembling of histological slides [8]. Histological evaluation of gut morphology was performed by one single evaluator recording villus height and width, crypt depth, and for calculating villus height:crypt depth ratio, and the proportion of proliferating cells to the total number of cells in the crypt using the Image JS tool [27] in ten pictures for each experimental unit (pig) according to the measurements described by Holanda and Kim [12]. Measurements of villus height and width were used to calculate the average mid-jejunal surface area for each villus by using the following formula [28]:

$$Surface\;area=2\pi \frac{villus\;width}{2}(villus\;height)+\pi \;{\left(\frac{villus\;width}{2}\right)}^{2}$$

### Statistical analysis

The statistical analysis was performed using the Mixed procedure of SAS 9.3 software (Cary, NC, USA). Each pig was considered as one experimental unit. Blocks (sex and initial body weight) were considered as random effects. Analyses of pre-planned contrasts between CON and MT as well as MT and MT+D were performed using the contrast statement. Results were considered statistically different for p<0.05 and considered a tendency for 0.05≤p<0.10. The design was based on the power test using previous studies conducted under similar conditions and objectives (n = 12 was minimum considering a large individual variation).

## RESULTS

The average mycotoxin contamination for the 3 dietary phases in CON samples was 1.2 mg/kg of deoxynivalenol, 0.21 mg/kg of fumonisin B1, and 0.15 mg/kg of zearalenone (Table 3). The average mycotoxin contamination for the 3 dietary phases in MT samples was 3.1 mg/kg of deoxynivalenol, 0.20 mg/kg of fumonisin B1, and 0.30 mg/kg of zearalenone.

**Figure 1:**
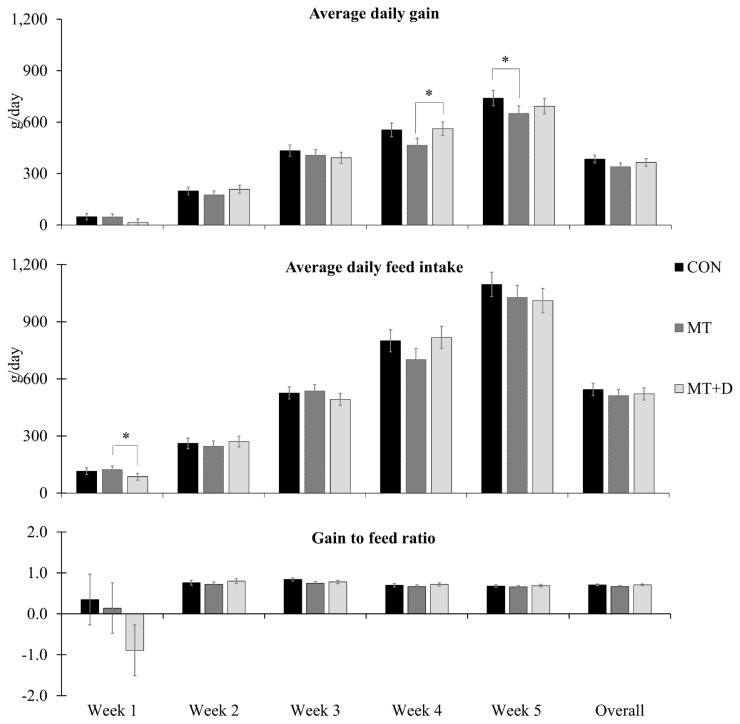
Average daily gain, average daily feed intake, and gain to feed ratio observed in weaned pigs consuming experimental diets with (MT) or without mycotoxins (CON) for 5 weeks. CON, control diet formulated with minimal deoxynivalenol contamination; MT, CON + 1.9 mg/kg of deoxynivalenol supplemented from mycotoxin contaminated corn DDGS; MT+D, MT + mycotoxin-detoxifying additive (0.2% of MegaFix; ICC, São Paulo, Brazil); and * 0.05≤p<0.10. Week 5 lasted 6 days to facilitate the sampling schedule.

There were no differences in pig body weight among pigs from experimental treatments (Table 4). Pigs fed MT+D tended to have increased (p = 0.099) averaged daily gain than pigs fed MT during week 4 (Figure 1). Pigs fed MT tended to present lower averaged daily gain during week 5 (p = 0.084) than pigs fed CON. Pigs fed MT+D tended to have lower (p = 0.099) averaged daily feed intake than pigs fed MT during week 1. There were no differences for gain to feed ratio or fecal score (Table 5) among pigs from experimental treatments.

**Figure 2:**
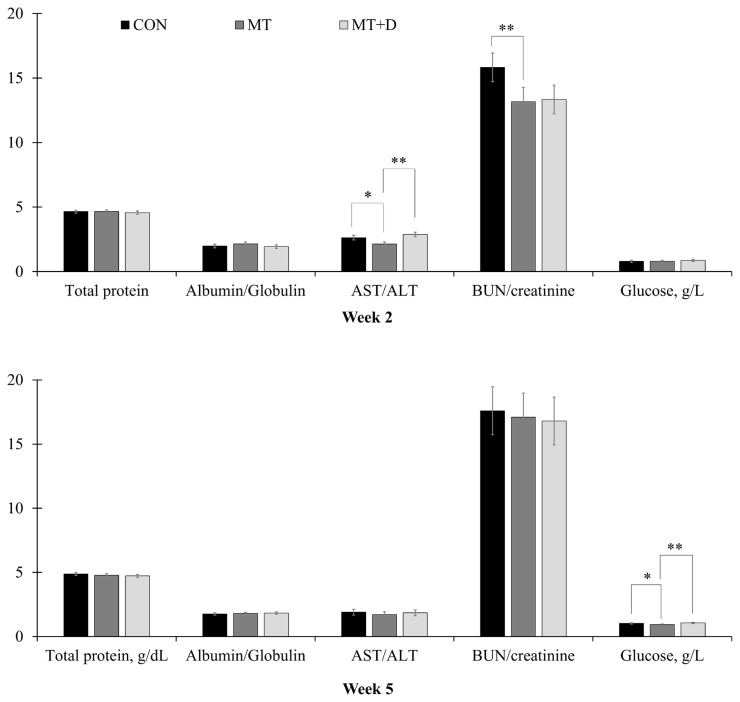
Serum variables observed in weaned pigs consuming experimental diets with (MT) or without mycotoxins (CON) by the end of week 2 and week 5. CON, control diet formulated with minimal deoxynivalenol contamination; MT, CON + 1.9 mg/kg of deoxynivalenol supplemented from mycotoxin contaminated corn DDGS; MT+D, MT + mycotoxin-detoxifying additive (0.2% of MegaFix; ICC, São Paulo, Brazil); AST, aspartate aminotransferase; ALT, alanine aminotransferase; BUN, blood urea nitrogen. * 0.05≤p<0.10; ** p<0.05.

By the end of week 2, pigs fed MT tended to have lower (p = 0.059) aspartate aminotransferase/alanine aminotransferase (AST/ALT) and lower (p<0.05) blood urea nitrogen/creatinine than pigs fed CON (Figure 2). Pigs fed MT+D had increased (p<0.05) AST/ALT than pigs fed MT. By the end of week 5, pigs fed MT tended to have reduced (p = 0.079) glucose in serum than pigs fed CON, whereas pigs fed MT+ D had increased (p<0.05) glucose levels than pigs fed MT. There were no differences in other variables assessed for biochemical profile among pigs from experimental treatments.

**Figure 3:**
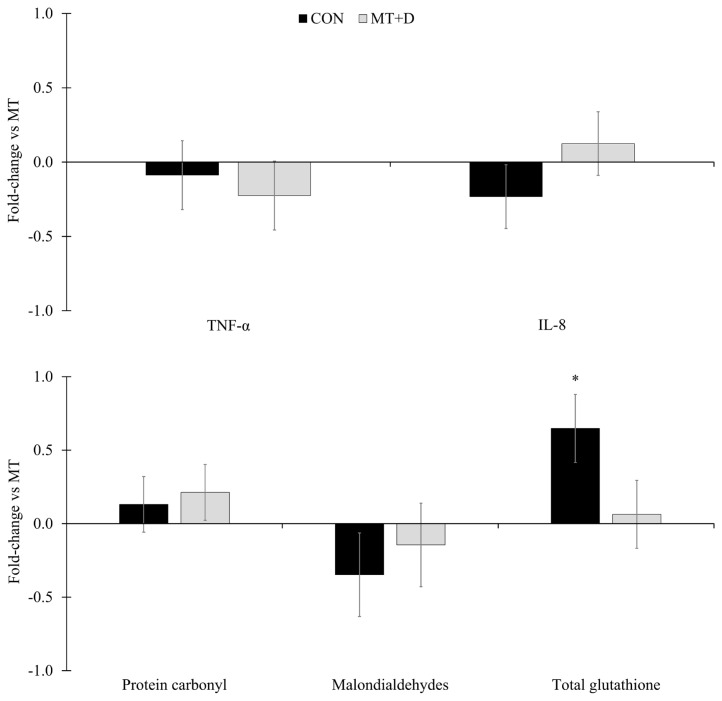
Inflammatory and oxidative stress markers in gut mucosa in weaned pigs consuming experimental diets with (MT) or without mycotoxins (CON) for 5 weeks expressed as fold-change relative to pigs fed MT. CON, control diet formulated with minimal deoxynivalenol contamination; MT, CON + 1.9 mg/kg of deoxynivalenol supplemented from mycotoxin contaminated corn DDGS; MT+D, MT + mycotoxin-detoxifying additive (0.2% of MegaFix; ICC, São Paulo, Brazil); TNF-α, tumor necrosis factor-alpha; IL-8, interleukin-8. * 0.05≤p<0.10.

Pigs fed CON tended to have higher (p = 0.057) concentration of total glutathione in mid-jejunal mucosa than pigs fed MT (Figure 3). There were no differences in tumor necrosis factor-alpha, interleukin 8, protein carbonyl, or malondialdehydes in mid-jejunal mucosa of pigs among experimental treatments. There were no differences in villus surface area, crypt depth, nor villus height to crypt depth ratio of pigs among experimental treatments (Table 6). Pigs fed MT+D had a lower (p<0.05) proportion of proliferating cells measured by Ki-67 staining in histological slides from mid-jejunum than pigs fed MT.

## DISCUSSION

As intended in the current study (supplemental 2 mg/kg), the MT diet had a supplemental 1.9 mg/kg of deoxynivalenol in comparison to CON. The intended concentration in MT aimed to surpass the guidelines proposed for this mycotoxin, for instance, 1 mg/kg in the United States [29] and 0.9 mg/kg in Europe [30] for growing pigs. Other mycotoxins frequently found as co-contaminants with deoxynivalenol [1] were detected, fumonisin B1 and zearalenone. Fumonisin B1 concentration did not exceed the guidance for growing pigs in the United States nor Europe [29,30] neither in CON nor MT. Zearalenone concentration in CON (0.15 mg/kg) and MT (0.30 mg/kg) exceeded the guidance levels in Europe of 0.1 mg/kg [30]. The European guidance levels include both young pigs (growing) and gilts (reproduction), but the latter group of animals was not the focus of the current study. Regarding growing animals, zearalenone effects include improved feed intake and growth [31] equivalent growth performance between challenged and unchallenged animals [32]. However, in naturally contaminated diets (along with other *Fusarium* toxins) a detrimental effect of zearalenone can be observed on pig growth [33]. Because of the controversial effects of zearalenone on pig growth in scientific publications, the effects of this mycotoxin will not be considered for further discussion.

The dietary intake of deoxynivalenol is known to reduce feed intake and gain of pigs [34]. The reduced feed intake may be caused by deoxynivalenol anorexigenic and emetic effects. It was described that deoxynivalenol mediated increase in cholecystokinin and, more significantly, in peptide YY is claimed as the mechanisms to result in reduced feed intake [35]. However, the current study could not demonstrate a consistent impairment in pig growth performance. Besides the reduction in averaged daily gain during week 5 in pigs fed MT in comparison to pigs fed CON, there was no further deoxynivalenol effect observed on growth performance. Pigs show reduced growth performance when fed from 1 to 3 mg/kg of deoxynivalenol [34]. In the current study, the CON diet had 1.2 mg/kg whereas the MT diet had 3.1 mg/kg of deoxynivalenol. Therefore, possibly pigs had some degree of growth impairment when fed the CON diet and only a moderate further decrease in growth performance could be noticed when performing the comparison among pigs fed MT and CON. Even though, despite the reduction in feed intake in pigs fed MT+D in comparison to pigs fed MT during week 1, pigs fed MT+D tended to have increased averaged daily gain than pigs fed MT during week 4. The improvement observed in growth performance in pigs challenged with deoxynivalenol could be due to the multiple component-approach (activated aluminosilicate, autolyzed yeast, probiotic yeast culture, calcarium marine algae powder, and enzyme complex) in the mycotoxin-detoxifying additive, as seen that deoxynivalenol is a mycotoxin that is known to be adsorbed or neutralized with difficulty [14].

The aluminosilicates have proven ability to adsorb mycotoxins, even though the deoxynivalenol adsorption is not as significant as in comparison to other mycotoxins [13,15]. The activated aluminosilicate, known as bentonite, was used in the current study to adsorb deoxynivalenol. As reviewed by Chaytor et al [14], the negative charge of AlO4 is responsible for the adsorbing ability towards mycotoxins, but as dependent on electrical interaction the adsorption of deoxynivalenol (non-polar) is not as substantial as for aflatoxins (polar). Therefore, mycotoxin detoxifiers targeting deoxynivalenol often show higher detoxifying capacity if multiple components are present both *in vitro* and *in vivo* [12,22].

The autolyzed yeast, used as a source of yeast cell wall, is the second form of adsorbent present in the composition of the mycotoxin-detoxifying additive. It differs from activated aluminosilicates for being organic, whereas activated aluminosilicates are inorganic adsorbents. Yeast- and algae-derived β-glucans can show higher deoxynivalenol adsorbing abilities than mineral adsorbents [15]. The β-glucans from the yeast cell wall may present around 7-times more adsorbing capacity than bentonites, being the highest in comparison to other organic adsorbents, like cellulose or a mixture of plan derivatives, microorganisms, and minerals [13].

The third ingredient in the mycotoxin-detoxifying additive is the probiotic yeast culture of *S. cerevisiae* at 2×10^7^ CFU/kg. The microbiota is able of microbial detoxification towards deoxynivalenol, but pig microbiota seems to have a lower detoxifying ability in comparison to ruminants and chickens, as observed *in vitro* [36]. Another *in vitro* study showed that pig microbiota from duodenum and jejunum have minimal detoxifying properties, whereas deoxynivalenol detoxification was observed for the microbiota from the cecum, colon, and rectum [37]. Nevertheless, deoxynivalenol is absorbed almost in its entirety in the stomach and proximal small intestine of the pig [38]. Consistently, during 24 hours after deoxynivalenol administration to pigs, either intravenous or intragastrical, showed that more than 90% of the initial dose was recovered without modifications [38]. Such findings may suggest that pigs are more vulnerable to deoxynivalenol in comparison to other species because of the rapid absorption of deoxynivalenol in the proximal gastrointestinal tract and its limited metabolism of the mycotoxin. The gastrointestinal microbiome of pigs may be modified when feeding deoxynivalenol contaminated diets [39], indicating that the gastrointestinal microbiome can be modulated to avoid deoxynivalenol toxicity. Thus, pig tolerance to deoxynivalenol can be improved by probiotic with deoxynivalenol-detoxifying properties, contained in the mycotoxin-detoxifying additive, to enhance microbial detoxification by promoting beneficial species. The microbial detoxification of deoxynivalenol is described to be mainly performed by anaerobic gram-positive bacteria in the gastrointestinal tract by conversion to the de-epoxide form [40]. Microbes with deoxynivalenol-detoxifying capacity are *Eubacteria*, *Anaerofilum*, *Collinsella*, *Bacillus*, and Clostridiales [40]. The use of yeast in the livestock feed chain [16], directed scientific efforts for its further use in livestock feed for mycotoxin detoxification. Under deoxynivalenol challenge, *S. cerevisiae* is able to reduce inflammation and oxidative stress and to increase cell survivability [17,41].

Modulating the gastrointestinal environment may help boost the adsorbing and detoxification properties of the mycotoxin-detoxifying additive in deoxynivalenol challenged pigs.

The calcarium marine algae powder in the mycotoxin-detoxifying additive is derived from *L. calcareum*. *In vitro*, *L. calcareum* has shown adsorption ability of zearalenone both in acidic and neutral pH [20]. In broilers challenged with mycotoxin diet, the addition of *L. calcareum* could recover animal weight gain [42] and prevent aflatoxicosis [21]. But there is a lack of studies regarding the adsorbability towards deoxynivalenol, especially concerning *in vivo* studies in pigs.

The enzymes present in the mycotoxin-detoxifying additive associated with the probiotic (*S. cerevisiae*) claim to have the ability to neutralize mycotoxins. The enzyme complex included in the mycotoxin-detoxifying additive formulation is composed of esterase, epoxide-reductase, and peptidase. The esterase activity has two main targets. First, as a detoxifier of zearalenone and fumonisins, which often are co-contaminants in animal feedstuff and feed due to being originated from *Fusarium* fungi [1]. As reviewed by Loi et al [19] the esterase has detoxifying activity by cleaving the lactone ring in zearalenone structure or breaking down the diester bonds in fumonisin B1. In addition, the reaction catalyzed by the esterase serves as an initial step for deoxynivalenol de-epoxidation. The deoxynivalenol in its masked naturally occurring forms, 3-acetyl-deoxynivalenol and 15-acetyl-deoxynivalenol, are converted to deoxynivalenol by the esterase activity [43]. The epoxide-reductase is responsible for the deoxynivalenol conversion to de-epoxy-deoxynivalenol, a non-toxic metabolite [18]. Besides, the epoxide-reductase activity relies on energy [36] or protein and amino acid [44] availability to perform the de-epoxidation reaction. Thus, the peptidase in the mycotoxin-detoxifying additive may provide the source of protein and amino acids required to enhance the epoxide-reductase activity. The slight improvement in averaged daily gain proportioned by feeding MT+D diet may indicate the detoxifying properties of the mycotoxin-detoxifying additive when included at 0.2% in diets contaminated with 3.1 mg/kg of deoxynivalenol. Comparable results were observed in growing pigs challenged with deoxynivalenol and zearalenone, where inclusion of a similar enzyme blend could enhance the growth performance of pigs [45].

In the current study, pigs fed MT had lower blood urea nitrogen/creatinine and AST/ALT than pigs fed CON, whereas pigs fed MT+D showed a recovery by increased AST/ALT than pigs fed MT. Likewise, pigs challenged with deoxynivalenol showed decreased blood urea nitrogen/creatinine and a mycotoxin detoxifier (composed of a mixture of clay, inactivated *S. cerevisiae* and its fermentation extracts, antioxidant, and botanicals) also caused an increase in AST/ALT in comparison to pigs fed diet contaminated with deoxynivalenol [24]. However, mice challenged with deoxynivalenol and zearalenone showed increased blood urea nitrogen due to mycotoxin impairment over kidney function [46]. Such outcomes may suggest that kidney damage was not significant in the current trial to lead to increased blood urea nitrogen. Instead, the deoxynivalenol inhibitory effect over protein synthesis, known as ribotoxic stress [4], could have a role in reducing protein metabolism and, thus, reducing the synthesis of AST and circulating levels of urea nitrogen [47].

The site of intestinal tissue collection was grounded on the fact that deoxynivalenol is mostly absorbed in the proximal gastrointestinal tract [38]. In addition, pigs fed deoxynivalenol presented a similar reduction on feed intake but had a greater reduction in body weight gain in comparison to pigs administered deoxynivalenol intraperitoneally [48], suggesting that deoxynivalenol may have local effects at the gastrointestinal tract that prevents nutrient digestibility and/or absorption. Indeed, the decrease in serum glucose observed in pigs fed MT in comparison to CON may be due to deoxynivalenol inhibition over sodium dependent glucose transporter 1 (SGLT1) in the brush border membrane in the small intestine, which limits glucose absorption. The deoxynivalenol-induced decrease in glucose uptake was demonstrated to be caused by impairing the expression and functions of the SGLT1 [49].

The lower proliferative rate in mid-jejunum observed in pigs fed MT+D in comparison to pigs fed MT in measuring Ki-67 positive cells proportion might suggest that MT+D diet could reduce deoxynivalenol toxicity in enterocytes and eventually reduce enterocyte death. Deoxynivalenol challenge causes increased expression of pro-inflammatory cytokines, such as interleukin-8 (IL-8), triggering cell apoptosis in the pig intestine [17,22]. Using *S. cerevisiae* as a probiotic source *in vitro* has shown the ability to reduce the expression of IL-8, tumor necrosis factor-alpha, and programmed cell death-related genes [41,50]. In a study with pig intestinal explants, the probiotic use of *S. cerevisiae* could counteract deoxynivalenol-induced inflammation and oxidative stress [17]. But there was no difference observed in inflammatory or immune markers measured in mid-jejunum in the current study. The only variable altered was total glutathione. The lower total glutathione presented by pigs fed MT in comparison to pigs fed CON may indicate that the concentration of 3.1 mg/kg of deoxynivalenol was surpassing the antioxidative capacity of pig mid-jejunum, depleting glutathione cell supply. De-oxynivalenol has inhibitory action over the vitamin C pathway, resulting in oxidative stress in pig intestine [17]. Vitamin C is important in glutathione regeneration to prevent oxidative stress, which may result in cell death [51,52].

In conclusion, feeding supplemental 1.9 mg/kg of deoxynivalenol reduced the average daily gain and debilitated hepatic and gut health by leakage of hepatic enzymes, impaired nitrogen metabolism, reducing glucose supply to the organism, and increasing oxidative stress in the gut. The mycotoxin-detoxifying additive mainly composed of bentonite (as adsorbent), calcarium algae powder (as adsorbent), enzymes (as mycotoxin inactivators), and *S. cerevisiae* (as a source of β-glucans—adsorbent and immune modulator—and as probiotic) enhanced hepatic health, glucose levels, and reduced proliferation of enterocytes in the crypt indicating attenuated gut damage in pigs fed diets contaminated with deoxynivalenol. However, the improvements promoted by the mycotoxin-detoxifying additive shown in gut health did not result in improved growth performance.

## Figures and Tables

**Table 1:** Concentrations of selected mycotoxins in clean corn DDGS or deoxynivalenol contaminated corn DDGS used for experimental diets

Mycotoxin (mg/kg)	Clean corn DDGS	Contaminated corn DDGS
Deoxynivalenol	2.620	7.643
Fumonisin B1	0.480	0.200
Zearalenone	0.213	2.417

Mycotoxin concentrations were measured at 37+ Lab (Alltech Inc., Nicholasville, KY, USA).

DDGS, dried distillers grains with solubles.

**Table 2:** Feed ingredients and calculated composition of experimental diets in a 3-phase feeding program fed to newly-weaned pigs for 5 weeks1)

Item	Phase 1 (wk 1)	Phase 2 (wk 2 and 3)	Phase 3 (wk 4 and 5)2)
Ingredient (%)			
Corn	14.72	31.12	43.22
Corn DDGS3)	22.00	22.00	22.00
Soybean meal	16.00	19.00	30.00
Whey permeate	20.00	10.00	0.00
Bakery meal	10.00	5.00	0.00
Poultry meal	6.00	4.00	0.00
Blood plasma	5.00	3.00	0.00
Fish meal	2.00	0.00	0.00
Poultry fat	2.00	3.00	2.00
Limestone	0.90	1.05	1.14
Dicalcium phosphate	0.00	0.50	0.70
L-lysine HCl	0.53	0.51	0.30
DL-methionine	0.15	0.12	0.02
L-threonine	0.10	0.10	0.01
Salt	0.22	0.22	0.22
Mineral premix	0.15	0.15	0.15
Vitamin premix	0.03	0.03	0.03
Titanium dioxide	0.00	0.00	0.05
Additive	0.204)	0.204)	0.204)
Chemical analysis			
Dry matter (%)	91.10	90.48	89.57
ME (kcal/kg)	3,471	3,480	3,391
SID lysine (%)	1.504	1.349	1.228
SID threonine (%)	0.876	0.800	0.732
SID tryptophan (%)	0.246	0.221	0.232
SID methionine +cysteine (%)	0.817	0.744	0.680
Ca (%)	0.849	0.799	0.711
STTD P (%)	0.469	0.407	0.334
Analyzed composition5)			
Dry matter (%)	94.04	93.43	93.01
Ca (%)	0.865	0.775	0.704
STTD P (%)	0.547	0.491	0.346

DDGS, dried distillers grains with solubles; ME, metabolizable energy; SID, standardized ileal digestible; STTD, standardized total tract digestible.

1) MegaFix (ICC, São Paulo, Brazil) was added to MT at 0.2% in all phases.

2) Week 5 lasted 6 days to facilitate the sampling schedule.

3) Either “clean” corn DDGS or deoxynivalenol contaminated corn DDGS (mycotoxin concentration: 7.643 mg/kg of feed of deoxynivalenol, 0.200 mg/kg of feed of fumonisin B1, and 2.417 mg/kg of feed of zearalenone) was used to formulate CON or MT diets, respectively.

4) Either corn (CON and MT) or the mycotoxin-detoxifying additive (MT+D) was added to diets.

5) The analyzed composition values are given on an as-fed basis.

**Table 3:** Concentrations of detected mycotoxins in experimental diets with (MT1)) or without mycotoxins (CON) fed to newly-weaned pigs for 5 weeks on a 3-phase feeding program2)

Mycotoxin (mg/kg of feed)	Phase 1 (wk 1)			Phase 2 (wk 2 and 3)			Phase 3 (wk 4 and 5)3)		
		
	CON4)	MT4)	MT+D4)	CON4)	MT4)	MT+D4)	CON4)	MT4)	MT+D4)
Deoxynivalenol	1.262	3.015	2.640	1.265	3.027	3.346	1.131	3.561	3.214
Fumonisin B1	0.2005)	0.2005)	0.2005)	0.2005)	0.2005)	0.2005)	0.238	0.203	0.214
Zearalenone	0.179	0.355	0.358	0.156	0.244	0.317	0.1005)	0.266	0.249

Mycotoxin concentrations were measured by liquid chromatography tandem mass spectrometry at the North Dakota State University Veterinary Diagnostic Laboratory (Fargo, ND, USA).

DDGS, dried distillers grains with solubles.

1) MT diets have 1.9 mg/kg of deoxynivalenol supplemented from mycotoxin contaminated corn DDGS.

2) MegaFix (ICC, São Paulo, Brazil) was added to MT at 0.2% in all phases to create another treatment, MT+D.

3) Week 5 lasted 6 days to facilitate the sampling schedule.

4) CON, control diet formulated with corn DDGS with minimal mycotoxin contamination; MT, diet formulated with corn DDGS with supplemental 5 mg/kg of deoxynivalenol contamination.

5) Non-detected mycotoxins had practical quantitation limits reported.

**Table 4:** Body weight of weaned pigs consuming experimental diets with (MT1)) or without mycotoxins (CON) for 5 weeks

Treatment	CON2)	MT2)	MT+D2)	SEM	p-value	

					CON vs MT	MT vs MT+D
Body weight (kg)						
Initial	8.20	8.20	8.22	0.54	0.956	0.878
Wk 1	8.55	8.52	8.33	0.52	0.923	0.424
Wk 2	9.94	9.76	9.79	0.59	0.616	0.929
Wk 3	12.98	12.60	12.53	0.69	0.543	0.909
Wk 4	16.86	15.86	16.47	0.85	0.265	0.496
Wk 53)	21.30	19.77	20.63	1.03	0.163	0.429

There was no animal mortality during the experimental period.

SEM, standard error of the mean.

1) MT and MT+D diets have 1.9 mg/kg of deoxynivalenol supplemented from mycotoxin contaminated corn DDGS.

2) CON, control diet formulated with corn DDGS with minimal mycotoxin contamination; MT, diet formulated with corn DDGS with supplemental 5 mg/kg of deoxynivalenol contamination; and MT+D, MT + MegaFix (ICC, São Paulo, Brazil) at 0.2%.

3) Week 5 lasted 6 days to facilitate the sampling schedule.

**Table 5:** Fecal score recorded in weaned pigs consuming experimental diets with (MT1)) or without mycotoxins (CON) at d 5, end of week 1, and end of week 2

Treatment	CON2)	MT2)	MT+D2)	SEM	p-value	

					CON vs MT	MT vs MT+D
Fecal score3)						
d 5	3.3	2.6	3.7	0.6	0.372	0.203
Wk 1	2.5	2.1	3.0	0.5	0.480	0.157
Wk 2	1.6	1.7	1.4	0.3	0.434	0.869

SEM, standard error of the mean.

1) MT and MT+D diets have 1.9 mg/kg of deoxynivalenol supplemented from mycotoxin contaminated corn DDGS.

2) CON, control diet formulated with corn DDGS with minimal mycotoxin contamination; MT, diet formulated with corn DDGS with supplemental 5mg/kg of deoxynivalenol contamination; MT+D, MT + MegaFix (ICC, São Paulo, Brazil) at 0.2%.

3) Fecal score was subjectively measured based on a 1 to 5 scale.

**Table 6:** Histological measurement from mid-jejunal morphology and immunohistochemistry in weaned pigs consuming diets with (MT1)) or without mycotoxins (CON) and diet with mycotoxins and feed additives by the end of week 5

Treatment	CON2)	MT2)	MT+D2)	SEM	p-value	

					CON vs MT	MT vs MT+D
Villus surface area ×10^3^ (μm^2^)	254.9	242.3	216.6	23.8	0.625	0.321
Crypt depth (CD) (μm)	256.5	249.5	229.9	12.3	0.622	0.174
Villus height/CD	1.64	1.79	1.62	0.24	0.425	0.381
Ki-673) (%)	29.43	27.23	21.62	1.92	0.191	0.002

SEM, standard error of the mean; DDGS, dried distillers grains with solubles.

1) MT and MT+D diets have 1.9 mg/kg of deoxynivalenol supplemented from mycotoxin contaminated corn DDGS.

2) CON, control diet formulated with corn DDGS with minimal mycotoxin contamination; MT, diet formulated with corn DDGS with supplemental 5mg/kg of deoxynivalenol contamination; and MT+D, MT + MegaFix (ICC, São Paulo, Brazil) at 0.2%.

3) Ki-67 counting is an estimate of the proliferative rate, calculated based on the proportion of cells positive to Ki-67 immunohistochemistry to the total cell number.

## References

[1] Gruber-Dorninger C, Jenkins T, Schatzmayr G (2019). Global mycotoxin occurrence in feed: a ten-year survey. Toxins.

[2] Lessard M, Savard C, Deschene K (2015). Impact of deoxynivalenol (DON) contaminated feed on intestinal integrity and immune response in swine. Food Chem Toxicol.

[3] Pestka JJ, Zhou HR, Moon Y, Chung YJ (2004). Cellular and molecular mechanisms for immune modulation by deoxynivalenol and other trichothecenes: unraveling a paradox. Toxicol Lett.

[4] Laskin JD, Heck DE, Laskin DL (2002). The ribotoxic stress response as a potential mechanism for MAP kinase activation in xenobiotic toxicity. Toxicol Sci.

[5] Dänicke S, Goyarts T, Döll S, Grove N, Spolders M, Flachowsky G (2006). Effects of the *Fusarium* toxin deoxynivalenol on tissue protein synthesis in pigs. Toxicol Lett.

[6] Weaver AC, See MT, Kim SW (2014). Protective effect of two yeast based feed additives on pigs chronically exposed to deoxynivalenol and zearalenone. Toxins.

[7] Weaver AC, Campbell JM, Crenshaw JD, Polo J, Kim SW (2014). Efficacy of dietary spray dried plasma protein to mitigate the negative effects on performance of pigs fed diets with corn naturally contaminated with multiple mycotoxins. J Anim Sci.

[8] Kim SW, Holanda DM, Gao X, Park I, Yiannikouris A (2019). Efficacy of a yeast cell wall extract to mitigate the effect of naturally co-occurring mycotoxins contaminating feed ingredients fed to young pigs: impact on gut health, microbiome, and growth. Toxins.

[9] Huwig A, Freimund S, Käppeli O, Dutler H (2001). Mycotoxin detoxication of animal feed by different adsorbents. Toxicol Lett.

[10] Frobose HL, Stephenson EW, Tokach MD (2017). Effects of potential detoxifying agents on growth performance and deoxynivalenol (DON) urinary balance characteristics of nursery pigs fed DON-contaminated wheat. J Anim Sci.

[11] Weaver AC, See MT, Hansen JA (2013). The use of feed additives to reduce the effects of aflatoxin and deoxynivalenol on pig growth, organ health and immune status during chronic exposure. Toxins.

[12] Holanda DM, Kim SW (2020). Efficacy of mycotoxin detoxifiers on health and growth of newly-weaned pigs under chronic dietary challenge of deoxynivalenol. Toxins.

[13] Kong C, Shin SY, Kim BG (2014). Evaluation of mycotoxin sequestering agents for aflatoxin and deoxynivalenol: an *in vitro* approach. Springerplus.

[14] Chaytor AC, Hansen JA, van Heugten E, See MT, Kim SW (2011). Occurrence and decontamination of mycotoxins in swine feed. Asian-Australas J Anim Sci.

[15] Sabater-Vilar M, Malekinejad H, Selman MHJ, van der Doelen MAM, Fink-Gremmels J (2007). *In vitro* assessment of adsorbents aiming to prevent deoxynivalenol and zearalenone mycotoxicoses. Mycopathologia.

[16] Kim SW (2010). Bio-fermentation technology to improve efficiency of swine nutrition. Asian-Australas J Anim Sci.

[17] Alassane-Kpembi I, Pinton P, Hupé JF (2018). *Saccharomyces cerevisiae* boulardii reduces the deoxynivalenol-induced alteration of the intestinal transcriptome. Toxins.

[18] Springler A, Hessenberger S, Reisinger N (2017). Deoxynivalenol and its metabolite deepoxy-deoxynivalenol: multi-parameter analysis for the evaluation of cytotoxicity and cellular effects. Mycotoxin Res.

[19] Loi M, Fanelli F, Liuzzi VC, Logrieco AF, Mulè G (2017). Mycotoxin biotransformation by native and commercial enzymes: present and future perspectives. Toxins.

[20] Keller K, Pereyra C, Almeida T, Cavaglieri L, Rosa C, Berthiller F (2010). Zearalenone adsorption by a commercial seeweed meal (Lithothamnium sp.). ISM conference 2009 worldwide mycotoxin reduction in food and feed chains.

[21] Perali C, Magnoli AP, Aronovich M, Da Rocha Rosa CA, Cavaglieri LR (2020). *Lithothamnium calcareum* (*Pallas*) *Areschoug* seaweed adsorbs aflatoxin B_1_
*in vitro* and improves broiler chicken’s performance. Mycotoxin Res.

[22] Park SH, Kim J, Kim D, Moon Y (2017). Mycotoxin detoxifiers attenuate deoxynivalenol-induced pro-inflammatory barrier insult in porcine enterocytes as an *in vitro* evaluation model of feed mycotoxin reduction. Toxicol In Vitro.

[23] Dersjant-Li Y, Verstegen MWA, Gerrits WJJ (2003). The impact of low concentrations of aflatoxin, deoxynivalenol or fumonisin in diets on growing pigs and poultry. Nutr Res Rev.

[24] Holanda DM, Yiannikouris A, Kim SW (2020). Investigation of the efficacy of a postbiotic yeast cell wall-based blend on newly-weaned pigs under a dietary challenge of multiple mycotoxins with emphasis on deoxynivalenol. Toxins.

[25] Committee on Nutrient Requirements of Swine, National Research Council (2012). Nutrition requirements of swine.

[26] Zhao Y, Kim SW (2020). Oxidative stress status and reproductive performance of sows during gestation and lactation under different thermal environments. Asian-Australas J Anim Sci.

[27] Almeida JS, Iriabho EE, Gorrepati VL (2012). ImageJS: personalized, participated, pervasive, and reproducible image bioinformatics in the web browser. J Pathol Inform.

[28] Rosero DS, Odle J, Moeser AJ, Boyd RD, van Heugten E (2015). Peroxidised dietary lipids impair intestinal function and morphology of the small intestine villi of nursery pigs in a dose-dependent manner. Br J Nutr.

[29] Food and Drug Administration (2005). Compliance program guidance manual.

[30] Official Journal of the European Union (2016). Commission recommendation (EU) 2016/13-19 of 29 July 2016 amending recommendation 2006/57-6/EC as regards deoxynivalenol, zearalenone and ochratoxin A in pet food (text with EEA relevance).

[31] Jiang SZ, Yang ZB, Yang WR (2010). Effects of feeding purified zearalenone contaminated diets with or without clay enterosorbent on growth, nutrient availability, and genital organs in post-weaning female pigs. Asian-Australas J Anim Sci.

[32] Jiang SZ, Yang ZB, Yang WR (2011). Effects of purified zearalenone on growth performance, organ size, serum metabolites, and oxidative stress in postweaning gilts. J Anim Sci.

[33] Swamy HVLN, Smith TK, MacDonald EJ, Karrow NA, Woodward B, Boermans HJ (2003). Effects of feeding a blend of grains naturally contaminated with *Fusarium* mycotoxins on growth and immunological measurements of starter pigs, and the efficacy of a polymeric glucomannan mycotoxin adsorbent. J Anim Sci.

[34] Marin S, Ramos AJ, Cano-Sancho G, Sanchis V (2013). Mycotoxins: occurrence, toxicology, and exposure assessment. Food Chem Toxicol.

[35] Flannery BM, Clark ES, Pestka JJ (2012). Anorexia induction by the trichothecene deoxynivalenol (vomitoxin) is mediated by the release of the gut satiety hormone peptide YY. Toxicol Sci.

[36] He P, Young LG, Forsberg C (1992). Microbial transformation of deoxynivalenol (vomitoxin). Appl Environ Microbiol.

[37] Kollarczik B, Gareis M, Hanelt M (1994). *In vitro* transformation of the *Fusarium* mycotoxins deoxynivalenol and zearalenone by the normal gut microflora of pigs. Nat Toxins.

[38] Prelusky DB, Hartin KE, Trenholm HL, Miller JD (1988). Pharmacokinetic fate of 14C-labeled deoxynivalenol in swine. Fundam Appl Toxicol.

[39] Reddy KE, Jeong JY, Song J (2018). Colon microbiome of pigs fed diet contaminated with commercial purified deoxynivalenol and zearalenone. Toxins.

[40] Yu H, Zhou T, Gong J (2010). Isolation of deoxynivalenol-transforming bacteria from the chicken intestines using the approach of PCR-DGGE guided microbial selection. BMC Microbiol.

[41] Chang C, Wang K, Zhou SN, Wang XD, Wu JE (2017). Protective effect of *Saccharomyces boulardii* on deoxynivalenol-induced injury of porcine macrophage via attenuating p38 MAPK signal pathway. Appl Biochem Biotechnol.

[42] Gonzalez NFG (2013). Aditivos anti-micotoxinas em dietas para frangos de corte [master’s thesis].

[43] Young JC, Zhou T, Yu H, Zhu H, Gong J (2007). Degradation of trichothecene mycotoxins by chicken intestinal microbes. Food Chem Toxicol.

[44] Ahad R, Zhou T, Lepp D, Pauls KP (2017). Microbial detoxification of eleven food and feed contaminating trichothecene mycotoxins. BMC Biotechnol.

[45] Cheng YH, Weng CF, Chen BJ, Chang MH (2006). Toxicity of different Fusarium mycotoxins on growth performance, immune responses and efficacy of a mycotoxin degrading enzyme in pigs. Anim Res.

[46] Liang Z, Ren Z, Gao S (2015). Individual and combined effects of deoxynivalenol and zearalenone on mouse kidney. Environ Toxicol Pharmacol.

[47] Webel DM, Finck BN, Baker DH, Johnson RW (1997). Time course of increased plasma cytokines, cortisol, and urea nitrogen in pigs following intraperitoneal injection of lipopolysaccharide. J Anim Sci.

[48] Prelusky DB (1997). Effect of intraperitoneal infusion of deoxynivalenol on feed consumption and weight gain in the pig. Nat Toxins.

[49] Awad WA, Vahjen W, Aschenbach JR, Zentek J (2011). A diet naturally contaminated with the *Fusarium* mycotoxin deoxynivalenol (DON) downregulates gene expression of glucose transporters in the intestine of broiler chickens. Livest Sci.

[50] Sougioultzis S, Simeonidis S, Bhaskar KR (2006). *Saccharomyces boulardii* produces a soluble anti-inflammatory factor that inhibits NF-κB-mediated IL-8 gene expression. Biochem Biophys Res Commun.

[51] Guaiquil VH, Vera JC, Golde DW (2001). Mechanism of vitamin C inhibition of cell death induced by oxidative stress in glutathione-depleted HL-60 cells. J Biol Chem.

[52] Niki E, Tsuchiya J, Tanimura R, Kamiya Y (1982). Regeneration of vitamin E from α-chromanoxyl radical by glutathione and vitamin C. Chem Lett.

